# Ibuprofen, other NSAIDs and COVID-19: a narrative review

**DOI:** 10.1007/s10787-023-01309-7

**Published:** 2023-08-21

**Authors:** William Laughey, Imran Lodhi, Graham Pennick, Lucinda Smart, Olutoba Sanni, Suneet Sandhu, Bruce Charlesworth

**Affiliations:** 1 Reckitt Health Care UK Ltd, Hull, UK; 2 Hull York Medical School, University of York, York, UK

**Keywords:** COVID-19, Ibuprofen, NSAIDs

## Abstract

At the start of the coronavirus disease 2019 (COVID-19) pandemic (March 2020), there was speculation that non-steroidal anti-inflammatory drugs (NSAIDs) such as ibuprofen, used to manage some of the symptoms of COVID-19, could increase the susceptibility to severe acute respiratory syndrome coronavirus 2 (SARS-CoV-2) infection and negatively impact clinical outcomes. In the absence of any robust mechanistic and clinical evidence, this speculation led to confusion about the safety of ibuprofen, contributing to the so-called ‘infodemic’ surrounding COVID-19. A wealth of evidence has been generated in subsequent years, and this narrative review aims to consider the body of in vitro and in vivo research, observational studies, systematic reviews and meta-analyses on the use of NSAIDs, including ibuprofen, in COVID-19. Overall, the direction of evidence supports that NSAIDs do not increase susceptibility to infection, nor worsen disease outcomes in patients with COVID-19. Neither do they impact the immune response to COVID-19 vaccines. There is no basis to limit the use of NSAIDs, and doing so may deprive patients of effective self-care measures to control symptoms.

## Introduction

**Fig. 1:**
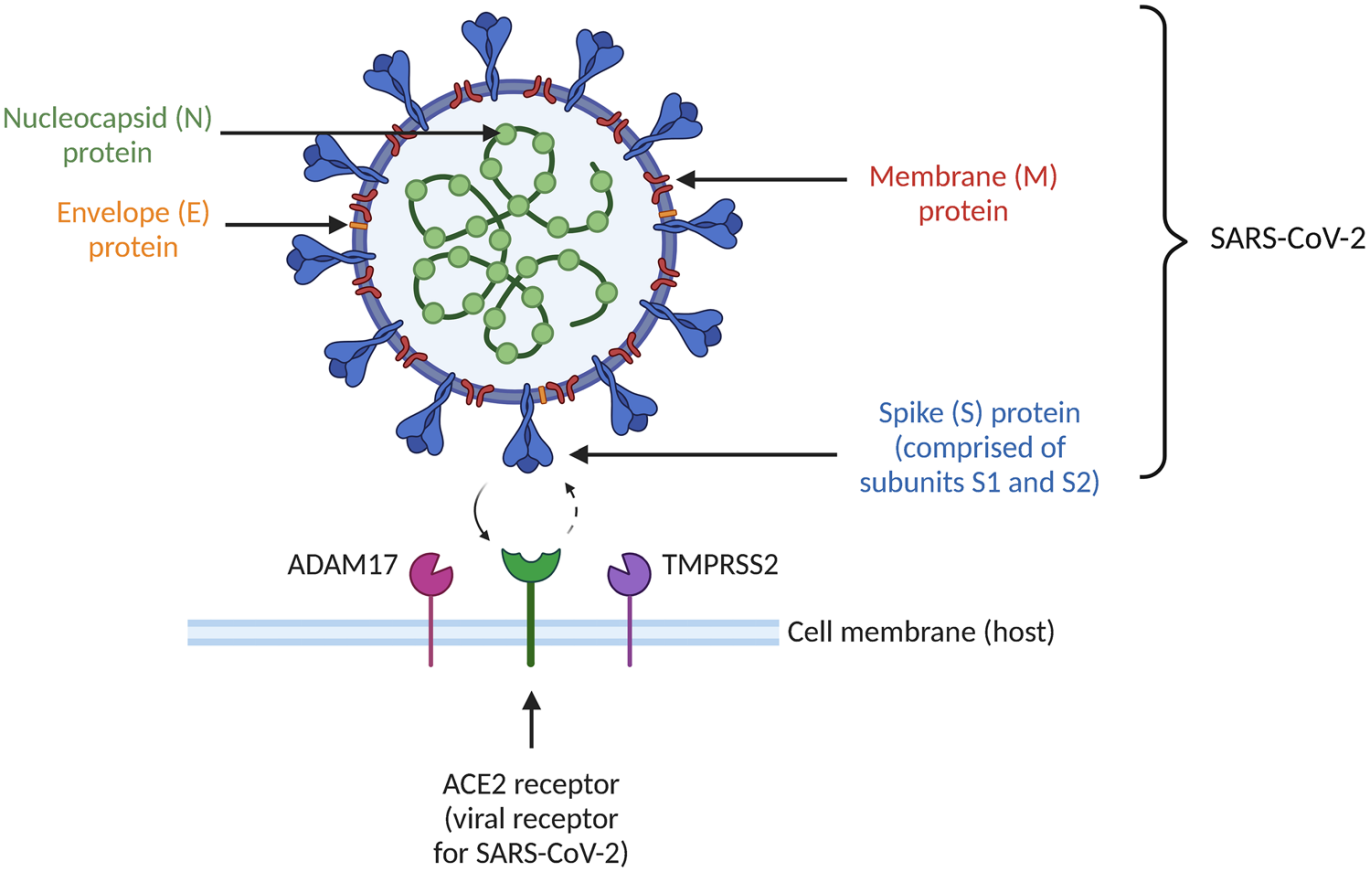
Structure of SARS-CoV-2 and the mechanism of entry into cells. *ACE2* angiotensin-converting enzyme 2, *ADAM17* A disintegrin metalloprotease domain 17, *TMPRSS2* transmembrane protease serine protease-2. Adapted from Smart et al. (2020)

The World Health Organisation (WHO) declared coronavirus disease 2019 (COVID-19), caused by severe acute respiratory syndrome coronavirus 2 (SARS-CoV-2), a pandemic on 11 March 2020 (World Health Organization 2020a). Early in the pandemic, non-steroidal anti-inflammatory drugs (NSAIDs), including ibuprofen, were used to manage some of the symptoms of COVID-19 (e.g. fever, cough, body aches and pains) due to their analgesic, anti-inflammatory or antipyretic effects (Kushner et al. 2022). On 11 March 2020, a letter published online in The Lancet highlighted that patients with comorbidities (diabetes or hypertension) who are treated with angiotensin-converting enzyme (ACE) inhibitors and angiotensin II type-I receptor blockers (ARBs) experience substantially increased expression of ACE2, the viral receptor for SARS-CoV-2 (Fig. 1). In addition, the letter speculated that ibuprofen use could increase expression of ACE2, facilitating COVID-19 infection and affecting disease outcomes. The letter cited no studies that directly linked ibuprofen use to ACE2 expression in patients (Fang et al. 2020); in fact, the finding that ibuprofen may upregulate ACE2 came from a single animal experiment in a non-COVID-related diabetic mouse model (Qiao et al. 2015).

Following the letter by Fang et al., the French Health Minister warned that taking anti-inflammatory medication (ibuprofen and cortisone in particular) could aggravate COVID-19 (Moore et al. 2020; Xaudiera and Cardenal 2020; Carius and Schauer 2020). Healthcare organisations and agencies across the world initially recommended against the use of ibuprofen, and although these statements were retracted just days later, the impact was profound (Moore et al. 2020; Carius and Schauer 2020). The reduction in ibuprofen prescriptions and over-the-counter purchases in some countries, coupled with global shortages of paracetamol, limited the choice of symptomatic treatments for patients (Moore et al. 2020; Smart et al. 2020; Zhou et al. 2022). Retrospectively, it has become clear that in the early stages of the pandemic, statements based on very little factual evidence could be greatly amplified. The WHO acknowledged that the COVID-19 pandemic has been accompanied by a so-called ‘infodemic’, given the exponential growth of information in a short space of time, making it difficult to find trustworthy sources and reliable guidance (World Health Organization 2020b).

The rapid availability of the COVID-19 vaccine substantially altered the course of the pandemic (Watson et al. 2022). Like other vaccines, COVID-19 vaccines are associated with side effects, including pain at the injection site, fever and fatigue. For decades, paracetamol and NSAIDs have been routinely used to alleviate such side effects (Ooi et al. 2022). However, there has also been speculation surrounding the impact of these treatment options on vaccine effectiveness, with some studies suggesting that paracetamol and NSAID use is associated with a blunted immune response (Ooi et al. 2022; Saleh et al. 2016; Prymula et al. 2009).

Since the start of the pandemic, there has been an abundance of in vitro and in vivo research, observational studies, systematic reviews and meta-analyses on the use of NSAIDs in COVID-19. In this narrative review, the following are considered: (1) in vitro and in vivo evidence evaluating the relationship between NSAIDs and COVID-19; (2) the clinical data covering the association between NSAIDs and the susceptibility to acquiring COVID-19 and disease outcomes, such as more serious illness and mortality and (3) the impact of antipyretics and analgesics on the immune response to COVID-19 vaccinations.

## Methods

### Search strategy

Since early in the pandemic (March 2020), an iterative process of compiling a database of papers relating to the potential effect of NSAIDs on clinical outcomes associated with COVID-19 was followed. Papers were identified through a process of recurring literature searches and manual searches of citations in published papers. In addition, pre-print servers such as MedRxiv were utilised during the height of the pandemic to capture further papers.

Most searches were conducted in Embase using the following search string: (‘coronavirus disease 2019’/exp OR ‘2019-ncov disease’ OR ‘2019-ncov infection’ OR ‘covid 19’ OR ‘covid 2019’ OR ‘covid19’ OR ‘wuhan coronavirus disease’ OR ‘wuhan coronavirus infection’ OR ‘coronavirus disease 2019’ OR ‘ncov 2019 disease’ OR ‘ncov 2019 infection’ OR ‘novel coronavirus 2019 disease’ OR ‘novel coronavirus 2019 infection’ OR ‘novel coronavirus disease 2019’ OR ‘novel coronavirus infection 2019’) AND (‘nonsteroid antiinflammatory agent’/exp OR ‘ibuprofen’/exp). In addition, PubMed was utilised to conduct additional searches from March 2020 to January 2023 using the following search string: (‘coronavirus disease 2019’) OR (‘COVID 19’) OR (‘COVID 2019’) OR (‘COVID2019’) OR (‘COVID19’) OR (‘novel coronavirus 2019 disease’) OR (‘novel coronavirus 2019 infection’) OR (‘novel coronavirus disease 2019’) OR (‘novel coronavirus infection 2019’) AND (‘nonsteroid antiinflammatory agent’) OR (‘NSAIDs’) OR (‘ibuprofen’).

### Patient and public involvement

No patients were involved in this study which used data only from published materials.

## Results

### In vitro and in vivo evidence evaluating the relationship between NSAIDs and COVID-19

Figure 1 shows the structure of SARS-CoV-2, a single-stranded RNA-enveloped virus, and the mechanism of entry into cells. The virus consists of four main structural proteins (envelope, membrane, nucleocapsid and spike). The spike protein consists of subunits S1 and S2, which play a vital role in virus–receptor bindings and cell fusion, respectively (Jiang et al. 2020; Beyerstedt et al. 2021). SARS-CoV-2 enters the host cell via the binding of the spike protein to the ACE2 receptor on the cell surface, which is abundantly expressed in the heart, kidneys and lungs (Beyerstedt et al. 2021). Transmembrane protease serine protease-2 (TMPRSS2) and A disintegrin metalloprotease domain 17 (ADAM17) of the host cell are intimately involved in the mechanisms of viral entry. ADAM17 is responsible for regulating ACE2 levels on the cell membrane, whilst TMPRSS2 cuts the S protein at the S1/S2 site for cellular uptake and viral replication (Beyerstedt et al. 2021; Smart et al. 2020).

Original theories about how NSAIDs could exacerbate COVID-19 included their hypothetical ability to upregulate ACE2, thus increasing the opportunity for SARS-CoV-2 virus to bind to host cells. These theories were based entirely on a specific diabetic mouse study (Qiao et al. 2015), and several mechanistic studies have since been published to assess the impact of NSAIDs on ACE2 regulation (Table 1).

**Table 1:** Summary of in vitro and in vivo studies

Reference	Study type	Study objective	Key findings
Chen et al. (2020)	In vitro (Calu-3 and Huh7.5 cell lines)	Relevance of COX-2/PGE2 signalling and inhibition by ibuprofen and meloxicam in vitro (maximum non-toxic dose)	SARS-CoV-2 infection induced COX-2 upregulation in human cells and mice Suppression of COX-2/PGE2 signalling by NSAIDs did not affect ACE2 expression, and therefore susceptibility to infection, in human cells and mice NSAID treatment did not affect SARS-CoV-2 entry or replication in human cells and mice
	In vivo (C57BL/6 J mice)	Relevance of COX-2/PGE2 signalling and inhibition by ibuprofen and meloxicam in vivo (ibuprofen: 30 mg/kg daily for 4 days; meloxicam: 1 mg/kg daily for 4 days)	
Valenzuela et al. (2021)	In vitro (human alveolar type-II pneumocyte cells)	Effect of ibuprofen on ACE2 levels, levels of spike protein internalisation, and ADAM17 and TMPRSS2 activities	Ibuprofen upregulated ACE2 expression 24 and 48 h after treatment Upregulation of ACE2 counteracted by ibuprofen-induced mechanisms that reduced SARS-CoV-2 spike protein internalisation, particularly by inhibition of ADAM17 and TMPRSS2 activities
	In vivo (healthy adult rats and rat model of metabolic syndrome [MetS: rats with obesity, hypertension, hyperglycaemia])	Effect of ibuprofen (40 mg/kg) on lung levels of ACE2 and ADAM17 and TMPRSS2 in healthy and MetS rats	In healthy and MetS rats, treatment with ibuprofen increased lung ACE2 expression and RAS activity
de Bruin et al. (2022)	In vitro (Caco-2 cell line)	Influence of ibuprofen, flurbiprofen, etoricoxib and paracetamol (increasing concentrations in the range of c_max_ plasma levels in humans) on the level of ACE2 mRNA/protein expression and activity, and influence on SARS-CoV-2 infection levels	All NSAIDs and paracetamol had no effect on ACE2 mRNA/protein expression and activity in the Caco-2 cell line Higher concentrations of ibuprofen and flurbiprofen reduced SARS-CoV-2 replication
	In vivo (C57BL/6 J mice)	ACE2 mRNA/protein levels and activity in the lung, heart and aorta of ibuprofen-treated mice (ibuprofen doses: 0 mg/kg, 50 mg/kg, 100 mg/kg and 200 mg/kg)	No upregulation of ACE2 mRNA/protein expression and activity in ibuprofen-treated mice compared with untreated mice Ibuprofen did not alter ACE2 activity

*ACE2* angiotensin-converting enzyme 2 receptor, *ADAM17* A disintegrin metalloprotease domain 17, *C*_*max*_ maximum concentration of a drug in the blood, cerebrospinal fluid or target organ, *COX-2* cyclooxygenase-2, *MetS* rat model of metabolic syndrome, *mRNA* messenger ribonucleic acid, *NSAID* non-steroidal anti-inflammatory drug, *PGE2* prostaglandin E2, *RAS* renin–angiotensin system, *SARS-CoV-2* severe acute respiratory syndrome coronavirus 2, *TMPRSS2* transmembrane protease serine protease-2

In vitro data from Chen et al. demonstrated that suppression of cyclooxygenase-2/prostaglandin E2 (COX-2/PGE2) signalling by ibuprofen and meloxicam did not affect ACE2 expression or SARS-CoV-2 entry or replication in Calu-3 and Huh7.5 cell lines (Chen et al. 2020). Similarly, de Bruin et al. found that NSAIDs (ibuprofen, flurbiprofen, etoricoxib) or paracetamol did not affect ACE2 mRNA and protein expression in the Caco-2 cell line; furthermore, ibuprofen and flurbiprofen had no effect on SARS-CoV-2 replication across most doses and reduced replication at higher doses (de Bruin et al. 2022). Regarding in vivo data, Chen et al. found no evidence that ibuprofen or meloxicam upregulate ACE2 expression in mice (Chen et al. 2020). Equally, de Bruin et al. did not observe increased ACE2 mRNA and protein expression in healthy ibuprofen-treated mice (de Bruin et al. 2022). On the other hand, a study by Valenzuela et al. noted that ibuprofen administration resulted in increased lung ACE2 protein expression and activity in healthy and metabolic syndrome (MetS) rats. In the same study, ibuprofen treatment of cultures of human alveolar type-II pneumocytes upregulated ACE2 expression; however, this was counteracted by an ibuprofen-induced decrease in SARS-CoV-2 spike protein internalisation by inhibition of ADAM17 and TMPRSS2 activities (Valenzuela et al. 2021).

Research by Chen et al. and de Bruin et al. demonstrates that there is no detrimental effect of the NSAIDs tested or paracetamol on ACE2 activity in vitro or in vivo. This contradicts the earlier hypotheses that NSAIDs may upregulate ACE2 and facilitate SARS-CoV-2 entry (Fang et al. 2020). Although Valenzuela et al. observed ibuprofen-induced upregulation of ACE2 in vitro and in vivo, ibuprofen decreased SARS-CoV-2 spike protein internalisation by inhibiting ADAM17 and TMPRSS2 activities in vitro, which indicates that upregulation of ACE2 does not necessarily correlate with increased viral entry (Valenzuela et al. 2021). The differing outcomes in ACE2 expression seen in vivo could be due to disparities in the experimental methods, such as the exposure time to ibuprofen and the use of different rodent models (Valenzuela et al. 2021; de Bruin et al. 2022).

An effective immune response against SARS-CoV-2 infection requires innate and adaptive immune systems. The innate immune response is the first line of defence, which occurs in any viral infection (Schultze and Aschenbrenner 2021). During this response, immune cells (e.g. macrophages, dendritic cells) produce interferons and pro-inflammatory cytokines. These go on to initiate the adaptive immune response where antibodies are produced that specifically target the virus. However, some patients with severe COVID-19 suffer from a ‘cytokine storm’, where the immune system causes an extreme and potentially lethal inflammatory response, with a sudden and uncontrolled release of pro-inflammatory cytokines (Montazersaheb et al. 2022; Ragab et al. 2020; Wong 2021). It has been suggested that the ‘cytokine storm’ plays a crucial role in the progression of SARS-CoV-2 infection and mortality and could be the major reason for multiple organ damage and an increased fatality rate in immunocompromised patients (Peng et al. 2021; Rabaan et al. 2021; Ragab et al. 2020). Perico et al. suggest that even early in the disease, the appearance of symptoms may mark a time point when there is already overactivation of the inflammatory response (Ruggenenti et al. 2022).

NSAIDs are frequently used for the relief of pain and inflammation, and it has been suggested that they could modulate SARS-CoV-2 infection and the host response to the virus (Chen et al. 2021). Chen et al. carried out a study in cell lines (Calu-3 and Huh7.5) and mice (C57BL/6 J and K18-hACE2) to assess how NSAID (ibuprofen or meloxicam) treatment may influence the cytokine and antibody response to SARS-CoV-2 infection (Chen et al. 2021). In human cell culture and mouse systems, suppression of COX-2 had no effect on ACE2 expression, viral entry or replication. However, in a mouse model of SARS-CoV-2 infection, NSAIDs impaired the production of pro-inflammatory cytokines and neutralising antibodies. Chen et al. suggested that NSAIDs could modulate COVID-19 severity by dampening neutralising antibodies and pro-inflammatory cytokines, which play an abundance of roles in controlling infection and driving immunopathology. Currently, it is unclear whether a dampened cytokine response is beneficial, detrimental or neutral in the setting of COVID-19, highlighting an area for further research (Chen et al. 2021).

### Clinical evidence assessing the association with NSAIDs and COVID-19

Since initial concerns that NSAIDs, including ibuprofen, could increase the risk of SARS-CoV-2 infection or COVID-19 severity (Fang et al. 2020), many observational studies, systematic reviews and meta-analyses have been conducted to assess whether NSAID use is associated with greater susceptibility to, or worsened outcomes in, COVID-19 (Table 2). There are also two randomised controlled trials (RCTs) to add to the evidence base (Recovery Collaborative Group 2022; Ravichandran et al. 2022).

**Table 2:** Summary of systematic reviews and meta-analyses

Reference	Country	Study design, data source	NSAIDs included	Sample size	Study aim	Outcomes included	Key findings
Moore et al. (2021)	Global	Systematic review and meta-analysis, PubMed MEDLINE (papers published in 2020 or 2021 [last search 1 March 2021])	NSAIDs	Sample size N/R (19 publications analysed)	Determine whether exposure to NSAIDs could increase the risk of testing positive for SARS-CoV-2 infection and whether this resulted in more severe disease outcomes	Hospital admission Intensive care unit admission Mechanical ventilation Mortality	No excess risk of SARS-CoV-2 positivity was observed NSAID exposure was not associated with an excess risk of hospital admission, death or severe outcomes in SARS-CoV-2-positive patients There was no increased risk of death with ibuprofen use
Zhou et al. (2022)	Global	Systematic review and meta-analysis, WHO COVID-19 database, Medline, Cochrane Library, Web of Science, EMBASE, China Biology Medicine Disc, China National Knowledge Infrastructure, and Wanfang database (search conducted from 1 January 2020 through 7 November 2021)	NSAIDs	4,867,795 (40 publications analysed)	Synthesise evidence on associations between the use of NSAIDs and adverse outcomes	Risk of SARS-CoV-2 infection Mortality Intensive care unit admission Mechanical ventilation	NSAID use: did not reduce mortality outcomes in patients with COVID-19 Not significantly associated with a higher risk of SARS-CoV-2 infection in patients with or without COVID-19, increased probability of ICU admission or increased requirement for mechanical ventilation or supplemental oxygen, compared with patients not using NSAIDs Ibuprofen use did not increase the risk of death
Zhao et al. (2022)	Global	Systematic review and meta-analysis, PubMed, EMBASE, Cochrane Library and MedRxiv databases (search conducted from December 2019 to January 2019)	NSAIDs	101,215 (25 publications analysed)	Examine the prevalence of NSAID use and associated COVID-19 risk, outcomes and safety	Prevalent use of NSAIDs Impact of NSAID use on COVID-19 risk Hospitalisation Mechanical ventilation Impact of NSAID use on severe COVID-19 infection Mortality	NSAID use before hospital admission or a diagnosis of COVID-19 was not associated with an increased risk of COVID-19, hospitalisation, mechanical ventilation or length of hospital stay However, NSAID use before hospital admission or a diagnosis of COVID-19 was associated with a decreased risk of severe COVID-19 and death

*COVID-19* coronavirus disease 2019, *COX* cyclooxygenase, *ICU* intensive care unit, *NSAIDs* non-steroidal anti-inflammatory drugs, *N/R* not reported, *SARS-CoV-2* severe acute respiratory syndrome coronavirus 2

In their narrative review, Kushner et al. report on 25 separate studies (24 publications in total) covering the period March 2020 to July 2021 (Kushner et al. 2022). Some of these studies draw from large datasets, such as Drew et al. (2.74 million participants) (Drew et al. 2021) and two studies by Wong et al. (Study 1: 2.5 million participants; Study 2: 1.7 million participants) (Wong et al. 2021). Most of the 25 studies considered NSAIDs as a class (18 studies) (Blanch-Rubió et al. 2020; Drew et al. 2021; Chandan et al. 2021; Wong et al. 2021; Hwang et al. 2020; Hasseli et al. 2021; Gianfrancesco et al. 2020; Jehi et al. 2020; Abu Esba et al. 2021; Lund et al. 2020; Park et al. 2021; Reese et al. 2021; Imam et al. 2020; Bruce et al. 2020; Drake et al. 2021; Jeong et al. 2021; Kow and Hasan 2021), some considered ibuprofen only or reported ibuprofen data separately (8 studies) (Wong et al. 2021; Rinott et al. 2020; Kragholm et al. 2020; Abu Esba et al. 2021; Castro et al. 2020; Choi et al. 2020; Samimagham et al. 2020), and some considered only aspirin or reported aspirin data separately (2 studies) (Drew et al. 2021; Chow et al. 2021).

Three studies considered whether NSAIDs affect susceptibility to acquiring COVID-19. The remainder focussed on whether the use of NSAIDs influenced the progression to more severe disease, for example, the requirement for oxygen, ventilation or intensive care and mortality. All three trials investigating the susceptibility to COVID-19 found no association with the use of NSAIDs and the risk of acquiring the disease (Blanch-Rubió et al. 2020; Chandan et al. 2021; Drew et al. 2021). For NSAIDs as a group, including ibuprofen and aspirin, of 22 studies that investigated the association between NSAIDs and outcomes in COVID-19, 13 reported no association (neither any improved nor worsened outcomes) (Chandan et al. 2021; Hwang et al. 2020; Hasseli et al. 2021; Gianfrancesco et al. 2020; Abu Esba et al. 2021; Lund et al. 2020; Park et al. 2021; Bruce et al. 2020; Drake et al. 2021; Rinott et al. 2020; Kragholm et al. 2020; Choi et al. 2020; Drew et al. 2021), 5 studies reported an association with improved outcomes (Wong et al. 2021; Imam et al. 2020; Castro et al. 2020; Chow et al. 2021) and 4 studies reported an association with worse outcomes (Reese et al. 2021; Jeong et al. 2021; Samimagham et al. 2020; Jehi et al. 2020), although for one of these studies, a later iteration of the data reported no association (Reese et al. 2022; Kushner et al. 2022).

For the eight studies that included specific ibuprofen data, six reported no association (Wong et al. 2021; Rinott et al. 2020; Kragholm et al. 2020; Abu Esba et al. 2021; Choi et al. 2020) with outcomes, one reported improved outcomes (Castro et al. 2020) and one reported worsened outcomes (Samimagham et al. 2020; Kushner et al. 2022). Kushner et al. concluded that, taken as a whole, this extensive body of research indicates there is no evidence to support a link with NSAIDs either to increasing susceptibility to acquiring COVID-19 or to worsening of disease outcomes (Kushner et al. 2022).

Drawing from much of the same evidence as Kushner et al., three meta-analyses, constituting the highest level of evidence currently available, have investigated the association of NSAIDs with COVID-19 (Moore et al. 2021; Zhou et al. 2022; Zhao et al. 2022). Moore et al. examined whether exposure to NSAIDs could increase the risk of testing positive to SARS-CoV-2 infection, and whether in such patients this resulted in more severe disease, as shown by hospital admission, admission to intensive care unit (ICU), mechanical ventilation or death. Of publications with analysable data, there was no increased risk of SARS-CoV-2 infection (odds ratio [OR] 0.86: 95% confidence interval [CI] 0.71–1.05), hospitalisation (OR 0.90; 95% CI 0.80–1.17), severe disease (OR 1.14; 95% CI 0.90–1.44) or mortality (OR 0.88; 95% CI 0.80–0.98) in populations exposed to NSAIDs as a class. Furthermore, in populations exposed to ibuprofen, there was no increased risk of mortality (OR 0.94; 95% CI 0.78–1.13) (Moore et al. 2021).

Zhou et al. aimed to synthesise evidence on the association between NSAID use and adverse events during COVID-19. Analyses were conducted on NSAIDs as a class for the main adverse outcomes, and subgroup analyses were also performed (stratified by NSAID type and population). For NSAIDs as a class, there was no significantly higher risk of SARS-CoV-2 infection (OR 0.96; 95% CI 0.86–1.07), ICU admission (OR 1.28; 95% CI 0.94–1.75), mechanical ventilation requirements (OR 1.11; 95% CI 0.79–1.54) or administration of supplemental oxygen (OR 0.80; 95% CI 0.52–1.24). The subgroup analysis revealed that ibuprofen was not associated with an increased risk of death compared with patients not using any NSAIDs (OR 1.09; 95% CI 0.50–2.39) (Zhou et al. 2022).

Zhao et al. assessed the prevalence of use, risk of disease and adverse outcomes of NSAIDs in patients with COVID-19 (Zhao et al. 2022). Analyses were conducted for the NSAID class, and subgroup analyses (stratified by NSAID type) were also performed. The prevalence of NSAID use was 19% amongst patients with COVID-19 and 18% when excluding a study that only covered in-hospital NSAID use. Prior exposure to NSAIDs (e.g. aspirin, ibuprofen and naproxen) was not associated with an increased risk of COVID-19 (adjusted odds ratio [aOR] 0.93; 95% CI 0.82–1.06), hospitalisation (aOR 1.06; 95% CI 0.76–1.48) or mechanical ventilation (aOR 0.71; 95% CI 0.47–1.06). In addition, prior NSAID use was associated with a decreased risk of severe COVID-19 (aOR 0.79; 95% CI 0.71–0.89) and death (aOR 0.68; 95% CI 0.52–0.89). Subgroup analyses showed that ibuprofen use did not significantly increase the risk of hospitalisation (OR 0.82; 95% CI 0.54–1.23), mechanical ventilation (OR 0.75; 95% CI 0.22–2.50), severe COVID-19 (OR 0.98; 95% CI 0.59–1.65) or death (OR 0.83; 95% CI 0.69–0.99) (Zhao et al. 2022).

All data described so far are at the level of observational studies. There are inherent limitations with observational data—particularly, susceptibility to confounding factors—which pose challenges for linking correlation with causation (Patorno et al. 2014). Arguably, such limitations have less relevance when data are generally indicating a lack of correlation, as is the case with NSAIDs and COVID-19. Randomisation is the most effective method for controlling for confounding factors, but when the premise begins with the hypothesis that a medication may be causing harm, as in this instance, it would be ethically questionable and practically challenging to recruit patients into a RCT. Observational designs are, therefore, the most appropriate evidence level, and they have the advantage of being able to recruit large numbers of participants in a relatively short space of time.

However, there are now two RCTs that have started with the hypothesis that NSAIDs may improve outcomes for patients with COVID-19 (RECOVERY Collaborative Group 2022; Ravichandran et al. 2022). Based on the premise that thromboembolic events are known complications in COVID-19, the RECOVERY trial randomised patients requiring hospitalisation to aspirin 150 mg daily plus usual care (*n* = 7351) vs. usual care alone (*n* = 7541) (RECOVERY Collaborative Group 2022). There was no significant difference in the primary endpoint of 28-day mortality (17% aspirin vs. 17% usual care; rate ratio 0.96 [95% CI 0.89–1.04]; *p* = 0.35). Patients allocated to aspirin had slightly fewer days in hospital (median 8 days vs. 9 days) and were slightly more likely to be discharged from hospital alive within 28 days (75% vs. 74%; rate ratio 1.06; 95% CI 1.02–1.10; *p* = 0·0062). Amongst those not on invasive mechanical ventilation at baseline, there was no significant difference in the proportion who progressed to invasive mechanical ventilation or death (21% vs. 22%; risk ratio 0.96; 95% CI 0.90–1.03; *p* = 0·23). The authors concluded that there was no compelling case for recommending aspirin in these patients (RECOVERY Collaborative Group 2022).

Beginning with the argument that indomethacin is known to have antiviral activity, in their open-label trial, Ravichandran et al. randomised patients to either indomethacin 75–150 mg daily plus usual care (*n* = 103) vs. paracetamol 2600 mg daily plus usual care (*n* = 107) (Ravichandran et al. 2022). The primary endpoint was deterioration in O_2_ levels to desaturation of SpO_2_ to 93% or below, which occurred in none of the indomethacin group but in 20 patients in the paracetamol group (*p* < 0.01). Furthermore, patients achieved symptomatic relief in half the time in the indomethacin group vs. the paracetamol group. Limitations include the relatively small sample size, lack of placebo control, the concomitant effects of other usual care medications, which included antivirals, and the open-label nature of the study. Nevertheless, these RCT findings point to a possible protective effect for indomethacin in addition to the benefits of symptom relief (Ravichandran et al. 2022).

The hypothesis that NSAIDs may possibly have a protective effect in COVID-19 if used regularly during the first few days of symptoms has recently been advanced by Perico et al., who propose a home treatment protocol that includes the use of NSAIDs, particularly COX-2-selective NSAIDs, in the first 3–4 days from the onset of symptoms (continued for longer if symptoms persist) (Perico et al. 2022). The authors cite mechanistic evidence around the benefits of mitigating inflammation early in the course of symptomatic disease and various exploratory cohort studies, including evidence around COX-2-selective NSAIDs (Perico et al. 2022). This evidence includes a retrospective observational matched-cohort study that assessed outcomes in patients with mild-to-moderate COVID-19. One cohort (recommended cohort, *n* = 90) was treated at home by family physicians at the onset of, or within a few days of the onset of, symptoms according to a treatment algorithm based on NSAIDs (priority for relatively selective COX-2 inhibitors [nimesulide or celecoxib] or other NSAIDs, corticosteroids, anticoagulants, antibiotics or oxygen therapy). In the control cohort (*n* = 90), none of the patients received relatively selective COX-2 inhibitors. The recommended treatment algorithm failed to accelerate recovery from major symptoms of COVID-19. However, other symptoms persisted in a lower number of patients in the recommended cohort compared with the control cohort (23.3% vs. 73.3%, respectively; *p* < 0.0001), fewer patients were hospitalised (2.2% vs. 14.4%, respectively; *p* = 0.0103) and the cumulative cost of hospitalisation was reduced by > 90%. The study is limited in its non-randomised design and the retrospective nature of the statistical analyses; however, the results could provide the background for designing future prospective trials in this context (Suter et al. 2021).

Currently, any evidence that the use of NSAIDs may be protective in COVID-19 remains at the level of generating possible hypotheses to explore in large, well-designed RCTs. Until this evidence is available, no firm conclusions around the possible protective benefits can be drawn. What can be said with certainty is that a wealth of evidence has accumulated on the associations between ibuprofen, other NSAIDs and COVID-19, and that this substantial body of evidence does not support the initial hypothesis that NSAIDs worsen outcomes in COVID-19. It is appropriate to state that we have moved from a lack of evidence of harm to evidence of a lack of harm. Ibuprofen and other NSAIDs do not increase susceptibility to COVID-19 and neither do they increase the risk of a worsening of the disease.

### Impact of antipyretics and analgesics on the immune response to COVID-19 vaccines

More than 2 billion doses of COVID-19 vaccine were administered worldwide within 6 months of the first approval (Ooi et al. 2022). However, despite the large number of doses delivered and the fact that COVID-19 vaccines are important to control the ongoing pandemic, some individuals were hesitant to receive the vaccine due to associated side effects (Ooi et al. 2022).

Post-vaccine symptoms, including pain at the injection site, headache, muscle pain, fever and fatigue, are relieved with antipyretic and analgesic medications, such as paracetamol or ibuprofen. Prior to COVID-19, there was a small body of evidence that the use of such medications could be associated with a blunted vaccine immune response, measured by antibody response to vaccine antigens (measured using antibody geometric mean concentrations [GMCs]). Prymula et al. found that 1 month after primary vaccination, antibody response to ten pneumococcal vaccine serotypes was significantly lower in the prophylaxis group. After booster vaccination, antibody response was lower for nine out of ten serotypes (Saleh et al. 2016; Prymula et al. 2009). It is important to note that these blunted immune responses to non-COVID-19 vaccines were only observed when antipyretic and analgesic medications were used prophylactically in advance of immunisation rather than as post-vaccination relief, and even then, there was no evidence that the reduction in antibody titres had a demonstratable effect on the clinical efficacy of vaccines (Saleh et al. 2016; Prymula et al. 2009).

There are limited data on the immunogenic impact of analgesic and antipyretic medications in relation to COVID-19 vaccines. However, emerging evidence is reassuring (Ooi et al. 2022). A protocol amendment in two of the five sites in a Phase 1/2, single-blind RCT allowed the administration of prophylactic paracetamol prior to administration of the Oxford/AstraZeneca COVID-19 vaccine. Participants receiving paracetamol had reduced adverse effects without affecting immunogenicity, based on antibody titres (Folegatti et al. 2020). Remaining data come from other COVID-19 vaccine trials in which participants were allowed to treat post-vaccination symptoms with analgesics and antipyretics (Ooi et al. 2022)—specific data on the medications used were not captured, but they were likely to be readily available options, such as paracetamol and ibuprofen. Phase 2/3 data on the Pfizer-BioNTech vaccine and Phase 3 data on the Janssen/J&J vaccine showed that younger recipients of the vaccines were more likely to use antipyretic or pain medications than older recipients, but despite this, vaccine efficacy remained stable across age groups. Furthermore, the fact that 25% of participants required medication for symptom relief did not prevent these vaccines from demonstrating excellent efficacy (Ooi et al. 2022). For the Pfizer-BioNTech vaccine, efficacy against confirmed COVID-19 was 95% for onset at ≥ 7 days after the second vaccine. For the Janssen/J&J vaccine, efficacy against severe–critical COVID-19 was 76.7% for onset at ≥ 14 days (Polack et al. 2020; Sadoff et al. 2021). These impressive levels of protection indicate that it is unlikely that the use of analgesics adversely affected vaccine efficacy. Although younger people were more likely to have used analgesics, the same high level of protection from immunisation was maintained (Laughey et al. 2022). Analgesic and antipyretic medications have been used for many years to manage vaccine-associated side effects and in doing so, reduce vaccine hesitancy; clinical trials of licenced COVID-19 vaccines used in combination with these medications do not suggest an impact on vaccine efficacy in the short term (Folegatti et al. 2020; Polack et al. 2020; Sadoff et al. 2021). Given there is no robust evidence to support the notion that analgesic and antipyretic medications impair the efficacy of COVID-19 vaccines, public health bodies continue to suggest the use of such medications, including ibuprofen, to relieve side effects associated with COVID-19 vaccination (Ooi et al. 2022).

## Discussion

Scientific efforts were greatly amplified during the pandemic, and never in history has there been so much new research accumulated so quickly on a single topic. However, in a time where pre-print servers were overrun with literature, it opened up room for error as the usual reviewing rigour was not applied in the interest of speed to publication (Chirico et al. 2020). That being said, sufficient time has now passed to reflect on the body of evidence accrued and to arrive at more accurate conclusions given the weight of the evidence now available.

The extensive body of literature reviewed in this paper supports the conclusion that the speculation around the safety of ibuprofen, which emerged in the first wave of infection, was inaccurate and misplaced. Historically, it can now be categorised as one of the contributors to the mass of misinformation and disinformation that comprised the so-called ‘infodemic’ or ‘misinfodemic’ of COVID-19. The term infodemic was first coined in the SARS outbreak of 2003(Tomes 2020) and is defined by WHO as an excess of information—including false and misleading information—during a disease outbreak (World Health Organization 2023).

The potential for infodemics to take root and flourish has increased with the proliferation of social media. Before the establishment of the internet, it was difficult for misinformation to gain traction. For example, during the 1918 Spanish flu pandemic, occasional rumours circulated that the contagion was a product of German biological warfare, but these never gained traction in print media and so were never widely publicised (Tomes and Parry 2022). In contrast, unscientific narratives about SARS-CoV-2 propagated through the virtual world more rapidly than the virus was spreading in the real one; for example, it is reported that one-fifth of Americans believed the microchip conspiracy theory, contributing to vaccine hesitancy (Pertwee et al. 2022).

Citing the potential to mislead and confuse, the WHO recognises that infodemics cause harm (Tomes and Parry 2022). Although it is appropriate for pandemic health communications to be open about the limits of scientific knowledge, the public loses confidence in medical messaging when health authorities release conflicting advice (Tomes and Parry 2022). The ibuprofen scare was one example of this. The tweet that catalysed it was from a health minister, and therefore carried medical authority. However, when first reported, it was recognised to be controversial given the lack of credible supporting evidence (CNN Health 2020), and within weeks, the FDA (US Food & Drug Administration 2020), the EMA (European Medicines Agency 2020) and the WHO (World Health Organization 2020c), amongst others, had released communications to clarify this lack of evidence. The impression given to the public was one of uncertainly and confusion, undermining confidence in the best approach to take to self-care.

Self-care forms a crucial part of managing respiratory viral infections; it reduces demands on health services (crucial in pandemics) and reduces unnecessary prescribing of antibiotics (Fokkens et al. 2020). The scare limited options as the public avoided ibuprofen and pharmacies sold out of paracetamol, and many patients were left without their usual symptomatic treatments (Moore et al. 2020; Smart et al. 2020; Zhou et al. 2022); meanwhile, medical cardiology societies were busy publishing statements to urge patients to continue to take medications such as ACE inhibitors for heart conditions after these medications were entangled in the same concerns regarding the theoretical upregulation of ACE2 as NSAIDs (American College of Cardiology 2020).

Whilst it is important to acknowledge that infodemics cause harm, it is even more pertinent to understand what can be done to prevent them, or at least mitigate their impact. In a recent WHO report, recommended strategies include the vigorous debunking of misinformation and the proactive and effective use of diverse media, including television, print and social media (Tomes and Parry 2022). As evident in our conflict-of-interest statement, all authors work for Reckitt, which manufactures and distributes products that were impacted during the pandemic, including Nurofen (based on ibuprofen), Dettol and Lysol. It was noted that no health organisations, in the initial phases at least, contacted Reckitt directly or via a trade association, despite marketing authorisation holders having a deep understanding of their medicinal products, access to pharmacovigilance data and an ability to effectively communicate important health-related messages to their consumers. This may represent a missed opportunity. Indeed, in future pandemics or health scares, there could be a case for industry to work more closely with governments and health bodies to help manage infodemics. Health-related industries are used to working with media outlets and have invested in building public trust in their brands, trust that is known to be related to brand loyalty (Lau and Lee 1999). As an illustration of the power of industry to communicate a clear health message in COVID-19, the Dettol hand-wash challenge, launched on Tik Tok to illustrate good hand-washing techniques, rapidly accumulated 125 billion views and at the time became the second most-viewed campaign in Tik Tok’s history (Financial Times 2020). Very few governmental or health agencies have this type of communication reach, and the potential for health-related industries to shape and magnify responsible health communications is considerable and largely underutilised.

Finally, the response of the scientific community and the timely accumulation of the high-quality NSAID and COVID-19 literature reviewed in this paper represent remarkable achievements. As an illustration of how quickly the science moved, a mere 18 months after the start of the NSAID scare, Moore et al. were already publishing their meta-analysis of 19 papers, which provided the first conclusive and reassuring evidence at the level of systematic review (Moore et al. 2021). Indeed, seen through an academic lens, whilst the scare around NSAIDs in COVID-19 deprived patients of effective symptom relief, it also opened the door to a rapid succession of rigorous research studies that have demonstrated the safety of the class in this model of infection.

## Conclusion

At the start of the COVID-19 pandemic, there were concerns that NSAIDs could increase susceptibility to infection or aggravate COVID-19 disease. Such hypotheses were theoretical, and the mechanism of ACE2 upregulation that was initially proposed was later largely disproved. Nevertheless, in the atmosphere of heightened anxiety that accompanied the arrival of a novel and highly contagious virus, these speculative concerns were amplified and led to a large-scale behavioural change in analgesic use, depriving patients of an effective drug to control pain and fever and contributing to the rapidly proliferating infodemic of COVID-19. Following these concerns, and progressing with remarkable efficiency, researchers have accumulated an extensive body of evidence—including in vitro and in vivo studies, observational clinical studies, RCTs and meta-analyses—that now confirms that NSAIDs are not associated with increased susceptibility to, nor with worsening outcomes in, COVID-19, and neither is there any evidence that they impair the effectiveness of COVID-19 vaccinations when used for the symptomatic relief of vaccine side effects. With the benefit of hindsight, there is the potential for health and hygiene companies to work more closely with health agencies to amplify responsible health messaging during pandemics and other health emergencies and to maintain trust in health authorities.

## Data Availability

No additional data are available.
